# Supplementary material for: Mortality in Antinuclear Antibody-Positive Patients with and Without Rheumatologic Immune-Related Disorders: A Large-Scale Population-Based Study
Source: Medicina (Kaunas). 2025 Jan 2;61(1):60. doi: 10.3390/medicina61010060 (PMC11767202; doi:10.3390/medicina61010060)
Supplement: Supplementary file 1 [file medicina-61-00060-s001.zip › medicina-3373428-supplementary.pdf]

Supplementary Materials:

Supplementary table S1. Clinical and Sociodemographic Factors Associated with All-Cause Mortality

**Variable**	**HR [a]**	**95% CI**	**Pv**	**Adjust. HR [b]**	**95% CI**	**Pv**
ANA Status	6.18	6.02-6.33	**<0.001**	4.62	4.50-4.74	**<0.001**
Age at ANA Test	1.09	1.08-1.09	**<0.001**	1.07	1.07-1.072	**<0.001**
Ethnicity, Jewish	1.40	1.34-1.46	**<0.001**	1.65	1.58-1.72	**<0.001**
Hyperlipidemia	2.82	2.74-2.91	**<0.001**	0.99	0.96-1.02	0.451
Hypertension	5.32	5.16-5.47	**<0.001**	1.39	1.35-1.44	**<0.001**
Malignancy	3.91	3.82-4.00	**<0.001**	1.90	1.86-1.95	**<0.001**
Autoimmune Disease	1.27	1.22-1.32	**<0.001**	0.83	0.79-0.86	**<0.001**

Note: Boldface type indicates $p<0.05$

HR = Hazard Ratio; CI = Confidence Interval; Pv = P-value.

[a] Univariate Cox Regression.

[b] Multivariable Cox Regression, adjusted to ANA status, age, ethnicity, hyperlipidemia, hypertension, malignancy, autoimmune disease.
